# EZH2: An analysis of a potential new tumor marker in high-risk localization of cutaneous squamous cell carcinomas

**DOI:** 10.3389/fonc.2024.1438021

**Published:** 2025-03-11

**Authors:** Cristian-Viktor Valea, Maurice Klein, Christian Hallermann, Hans-Joachim Schulze, Jan-Dirk Raguse, Kai Wermker

**Affiliations:** ^1^ AllDent Zahnzentrum Leipzig GmbH, Leipzig, Germany; ^2^ Department of Oral and Maxillofacial Surgery, School of Medicine, University Hospital RWTH Aachen, Aachen, Germany; ^3^ Laboratory of Dermatopathology and Pathology Hamburg-Niendorf, Hamburg, Germany; ^4^ Department of Dermatology and Histopathology, Fachklinik Hornheide, Münster, Germany; ^5^ Department of Oral & Maxillofacial Surgery, Fachklinik Hornheide, Münster, Germany; ^6^ Department of Oral & Cranio-Maxillofacial Surgery, Klinikum, Osnabrück GmbH, Osnabrück, Germany

**Keywords:** EZH2, prognostic factor, biomarker, lymph node metastasis, tumor marker, SCC, squamous cell carcinoma

## Abstract

**Background:**

Enhancer of zeste homolog 2 (EZH2) is a transcriptional enzyme implicated in tumor development and is often correlated to poor patient outcomes in various malignancies. The study evaluated various methods for EZH2 expression in lip and ear squamous cell carcinomas (LSCC, ESCC) by matching patients with and without lymph node metastasis (LNM) and further analysis of clinical outcome parameters. EZH2 inhibition therapy has shown promising results in multiple cancer entities, with ongoing research exploring its potential in other malignancies. This approach may also be applicable to high-risk LSCC and ESCC.

**Methods:**

A total of 122 patients who had been surgically treated for LSCC and ESCC were selected to form LNM-positive and LNM-negative matched pairs. EZH2 expression has been examined after immunostaining of the tumor tissue with EZH2-antibodies and quantified as extent, intensity, and score. Pursuing the clinical benefit, we analyzed three different EZH2-score approaches to determine aberrations in EZH2 expression.

**Results:**

While the overall EZH2 extent did not correlate with clinical outcome, the EZH2-intensity and -score was lower in patients who developed a local relapse or distant metastasis (DM). High EZH2-scores correlated with increasing grading, pN-, and American Joint Committee on Cancer-stage. Overall, the carcinoma tissue samples showed a high expression of EZH2 (mean expression > 60%).

**Conclusion:**

The hypothesis of the predictive prognostic contribution of EZH2 in clinical decisions regarding the occurrence of LNM was not substantiated by our data. Nevertheless, the elevated expression of EZH2 we have observed in our findings could be utilized as a pretherapeutic assessment prior to targeted therapies with tazemetostat. Subsequent research should substantiate this hypothesis.

## 1 Introduction

The predominant skin neoplasia is the nonmelanoma skin cancer (NMSC), which includes cutaneous squamous cell carcinoma (cSCC). Over 75% of the cSCCs are recorded in the head and neck region (1). The NMSC frequently affects light-skinned populations, with increasing incidence in many territories (2–4).

The identification of early genetic pathological variations, recognition of tumor suppressor genes, and proto-oncogenes contributes to furthering cancer therapy. A conscientiously and accurate clinical and histological examination is favorable, as well as a secure diagnosis at an early stage, which can result in reduced morbidity, better outcomes, and higher survival rates (5, 6).

Despite multimodal therapy, lymph node invasion contributes unfavorably for disease recurrence and progression, and is therefore clinically compelling (7–12). The 10-year survival rates of cSCCs are reduced <20% when associated with regional lymph node metastasis (LNM) and <10% with distant metastasis (DM) (13).

Many variables are acknowledged predictors for outcome and survival of cSCC-patients: TNM-stage, tumor depth, grading, desmoplasia, perineural growth, blood and lymph vessel invasion, immunosuppression, age, and others. Localization, inflammation, chronic wounds, and some markers are considered but remain uncertain and further research regarding tumor spread and malignancy risk is required (1, 8, 11, 14). Squamous cell carcinoma of the lip (LSCC) and squamous cell carcinoma of the ear (ESCC) are often referred to as high-risk localizations with increased risk of recurrence, metastasis (13), and influences on disease-specific survival (7).

In high-risk localizations of cSCC, tumor markers can help to assess the tumor aggressiveness (14). A tumor marker with such potential could be the Enhancer of Zeste Homolog 2 protein (EZH2).

EZH2 is a methyltransferase enzyme, functioning as core component of the Polycomb repressive Complex 2. EZH2 catalyzes the methyl-group addition to histone H3 at Lys27 (H3K27) and it contributes to epigenetic regulation of physiological cell differentiation and metabolism (15). EZH2 aberrations and polymorphisms associate with genetic mutations and therefore pathogenesis (16–18).

Recent studies have indicated that EZH2-overexpression correlates with tumorigenesis, progression, invasion, and dissemination in several malignancies including cSCC (16, 19–22). Novel studies have investigated the role of EZH2 as a prognostic factor and as a therapeutic target in multiple cancer entities including cSCC (16, 18, 20, 23–27). Some reports indicate that EZH2-expression interacts with chemotherapy efficiency (19, 20, 28, 29). The first EZH2 pathway-inhibitor tazemetostat was approved in 2020 by the FDA for treating epithelioid sarcoma. While current studies focus on the oncogenic role of EZH2, there is evidence for a tumor suppressive function of EZH2 in cancer immunity (18, 20). Baquero et al. (30) described a more invasive growth of the oral squamous cell carcinoma (SCC) after EZH2 knockdown. Analyzing the role of EZH2 in various tumor types is therefore clinically relevant.

The aim of this study was to determine whether the tumor marker EZH2 can be used to evaluate the potential aggressiveness of high-risk localizations of cSCC and to analyze various evaluation methods for its expression. These results could be the basis for the possible use of tazemetostat in advanced patients in follow-up studies.

A particular focus was placed on LNM prediction as well as disease progression, local relapse, pN-stage, DM, and disease related death (DRD) as tumor follow-up parameters. Different evaluation methods of EZH2 were applied and correlated with clinical pathological follow up values.

## 2 Methods

### 2.1 Patients

Patients with histologically confirmed squamous cell carcinoma (SCC) of the lip and ear who were surgically treated between 2004 and 2015 were retrospectively identified from our institutional database. Data acquisition, anonymization, and analysis were conducted according to data protection regulations. Inclusion criteria were SCC of the lip (including vermillion and mucosa) or the external ear (ICD-10: C44.2/C44.0/C.44.8/C00), having undergone surgery, histologically confirmed carcinoma, and the availability of complete patient information and follow-up data in our database. All patients received proper staging prior to surgery according to prevailing German guidelines (31). Exclusion criteria were patients diagnosed with multiple head and neck squamous cell carcinomas (HNSCC), or those who had previously undergone neck dissection. The patients were selected in a systematic manner to ensure the inclusion of the largest possible number of cases with the fewest possible differences in parameters for further matching, thus creating the primary collective. Furthermore, after an initial selection of 679 eligible patients with lip and ear SCC, we composed matched groups, resulting in a total of 122 patients, divided into metastasis and non-metastasis groups. The matching of the pairs included parameters that affect disease-specific survival, such as tumor localization, pT, tumor invasion variables (tumor depth, perineural growth, cartilage invasion), tumor grading, age at first diagnosis, gender, immunosuppression and comorbidities. Therefore, patients with an insufficient data set were excluded. The inclusion of further comorbidities or matching of specific comorbidities would encompass an effect on overall survival and result in a more heterogenous group division. The comorbidities were documented in our database but incorporated into the statistical analysis as a dichotomous variable, with or without comorbidities. The Lip-LN0 (n = 29) included patients with lip carcinoma who had not developed locoregional LNM during the follow-up period, while the Lip-LN+ group (n = 29) consisted of patients with lip carcinoma who initially had, or developed during the follow-up period, locoregional LNM. A similar distribution was conducted in the Ear-LN0 (n = 32) group, with patients with ear carcinoma who had not developed locoregional LNM during the follow-up period. The last group, Ear-LN+ (n = 32), included patients with ear carcinoma who initially had, or developed during the follow-up period, locoregional LNM.

The patient database of our institute served for multiple immunohistochemical biomarker research studies. This study shared its patient cohort with Klein et al. (12, 14).

### 2.2 Immunohistochemistry

After data censoring, slides (4 µm thick, Microm^®^ microtome, Walldorf, Germany) were obtained from paraffin-embedded tissue blocks (Vogel™ embedding medium VO-5-1001, Fernwald, Germany). EZH2-expression was examined according to the manufacturer’s guidelines (Dako REAL™ Detection System, Alkaline Phosphatase/RED, Mouse, Code-No. K5005, Waldbronn, Germany). The objects were incubated overnight for 12h at 37°C to achieve better adhesion between tissue samples and slides (Thermo Scientific™, Waltham, USA).

After deparaffinization and rehydration to buffer (xylene bath, placed in descending ethanol concentrations of 100%, 90%, and 70%, and dual aqua destillata rinsing) the slides underwent the heat-induced epitope retrieval protocol (HIER) to unmask the antigen targets for the antibodies. They were then incubated with Dako REAL™ Target Retrieval Solution, pH 9.0, Code-No. S2367, for 20’ at 90°C, with aqua destillata rinsing. Surface tension was reduced by rinsing with Dako REAL™ Wash Buffer, Code-No. S3006. The immunostaining process consisted of three steps which were conducted using the Dako REAL™ Detection System K5005.

The slides were incubated with anti-EZH2 antibodies for 20’ (Cell Marque™ EZH2 Mouse Monoclonal Antibody, Key-Code CMC41521000, Rocklin, USA). A second incubation of the sections with Dako REAL™ Link Biotinylated Secondary Antibodies (AB2) was conducted for 15’. The last incubation was with Dako REAL™ Streptavidin Alkaline Phosphatase for 15’.

The reaction was made visible using Dako REAL™ Chromogen Red (8’ of exposure), resulting in nuclear staining. Patient sections were washed and counterstained with hematoxylin (Dako REAL™ Hematoxylin, Code-No. S2020. cSCC-tissue was used for positive controls for EZH2-staining. To avoid nonspecific staining, phosphate-buffered saline was used as the negative control instead of primary antibodies.

### 2.3 Semi-quantitative evaluation of EZH2

The slides were evaluated with a polarizing microscope (Olympus BX51, 40x-lens, Hamburg, Germany) and analyzed by two examiners (CV and CH) from the institute research group, who were blinded to the clinical and histopathological data. If discrepancies occurred, the observers revised the slides simultaneously to achieve a unanimous result. An Olympus UC30 microscope camera was used for digital documentation. Photo editing was performed with Olympus cellSens Entry 1.17^©^.

For each slide, five random high-power fields with intensive immunostaining of the tumor tissue were selected for examination, following selection of five random cell groups of ten tumor cells each.

Positive EZH2-expression was defined by identifying different staining intensities in the cell nuclei. Within cell groups, we counted the EZH2-positive cells to detect staining extent percentage. Ultimately, cell observation was summed up to 250 random cells for each slide.

**Figure 1:**
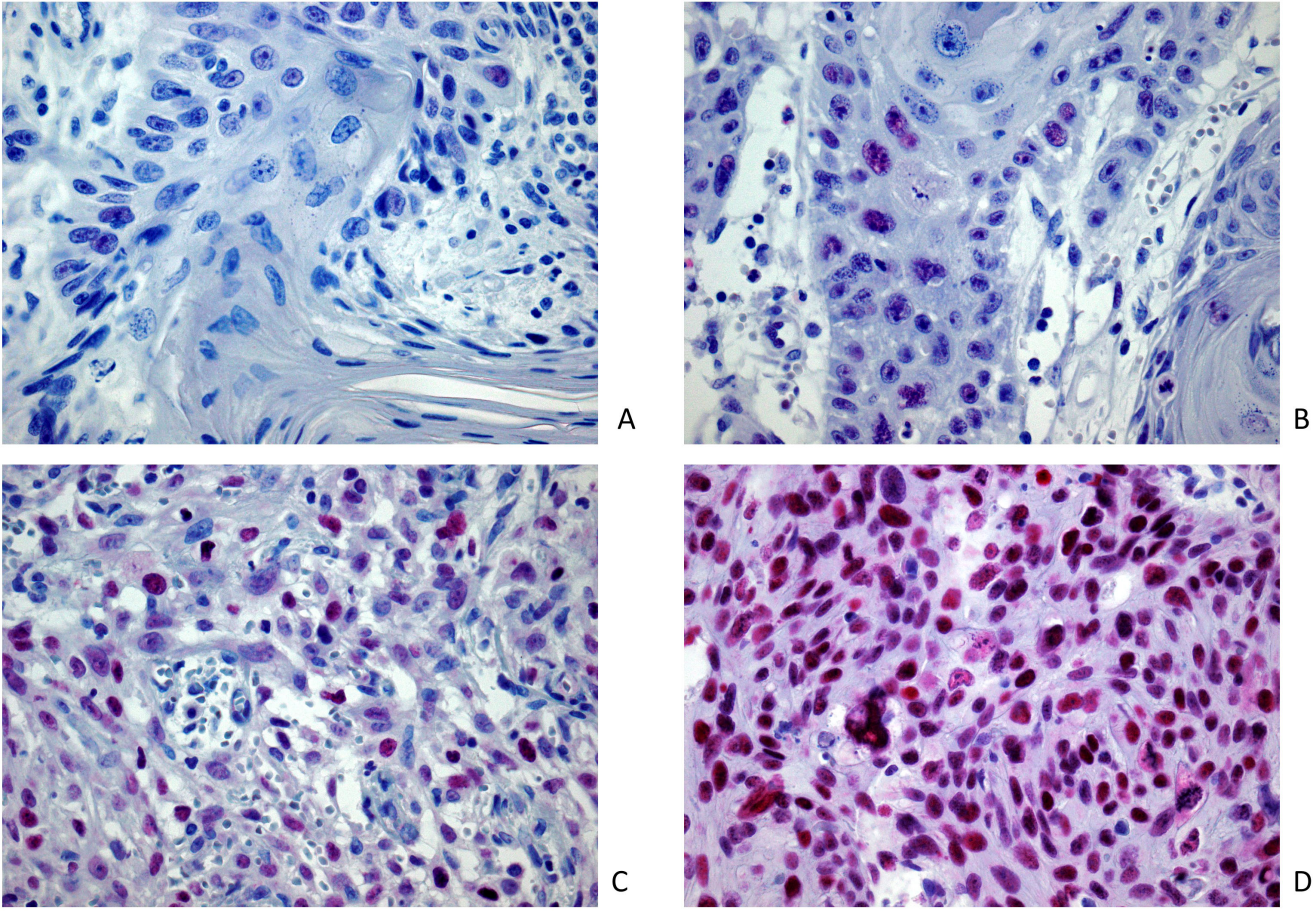
Image samples of positive EZH2 expression and intensity classification after immunohistochemical EZH2 staining (magnification 400x): **(A)** lowest registered staining intensity (M = 0.6); positive expression extent (n = 6.8%); **(B)** weak staining intensity (M = 1.0); positive expression extent (n = 66.0%); **(C)** moderate staining intensity (M = 2.0); positive expression extent (n = 56.0%); **(D)** strong staining intensity (M = 2.96); positive expression extent (n = 90.4%).

Staining intensity was graded for each cell-group and classified as: 0 (no staining), 1 (weak), 2 (moderate), and 3 (strong). **Figure 1** shows an overview of the immunohistochemical EZH2 staining.

### 2.4 EZH2 expression analysis as an EZH2 score model

While some studies have analyzed EZH2 expression *in situ* by amount (n=%) of positive stained cells (22, 25), the variability and importance of intensity of the staining has been noted and discussed (32, 33). We therefore developed three EZH2-scores based on previously validated scoring systems, also incorporating extent and intensity of positive stained cells (19, 23, 28). The purpose was to determine if EZH2 expression can be declared as a EZH2 score model with prognostic usability. The EZH2 extent category is defined by the proportion of positive stained cells, regardless of the intensity (n=%). The EZH2 expression scores were defined as follows: EZH2-score-I = multiplication product of staining extent category (n = 1–4, where 1 = <25%; 2 = 25–50%; 3 = 50–75%; and 4 = >75%) and mean intensity score (n = 0–3), obtaining an overall score from 0–12. EZH2-score-II = multiplication product of staining extent category (n = 1–3, where 1 = <35%; 2 = 35–70%; and 3 = >75%) and mean intensity score (n = 0–3) obtaining an overall score from 0–9. EZH2-score-III multiplied the percentage of EZH2-positive stained cells (n = 0–100%) with the mean staining intensity score (n = 0–3). In order to ensure the comprehensive analysis of the exact outcome of EZH2 stained cells (n=%), defined as expression extent, we have incorporated a third scoring model. To preserve the numerical model of an ordinal scale, we declared EZH2-score-III = $\frac{(\%extent*\;\bar{\text{x}}\text{ intensity score)}}{30}$ to obtain an overall score from 0–10.

### 2.5 Statistical analysis

The significance level for statistical hypothesis testing was defined as 5% cutoff (p-value <0.05, type I error rate <5%). IBM SPSS Statistics version 18.0 (SPSS Inc., Chicago, RRID: SCR_016479) was used for the statistical analyses. Categorical variables were organized in contingency tables and analyzed using the chi-square test of independence. The chi-square test is applied under the common assumption that the frequency distribution across a specific subgroup in a contingency table is greater than five in more than 80% of its cells. If this criterion was not met, the Fisher’s exact test was applied for 2x2 cross-tables, and for cross-tables greater than 2x2, the likelihood ratio, also known as the maximum likelihood radio chi-squared test, was employed. To assess the presence of a linear trend within a contingency table, the linear-by-linear association test was utilized for the analysis of EZH2 scores and tumor grading. The Mann-Whitney-U-Test was used for non-parametric evaluations of non-normally distributed data. This included the analysis of parameters such as LNM, DRD, disease progression, DM and local relapse. For ordinal scaled variables, such as pT, pN, staging of the American Join Committee on Cancer (AJCC) and tumor grading, the Kruskal-Wallis test was used in addition to the Mann-Whitney-U-Test for non-parametric analysis. The EZH2 scores were first analyzed using Levene’s test for Equality of Variances and subgroup evaluation proceeded using a two-sided Student’s t-test as independent samples.

## 3 Results

### 3.1 Study population

Our study included 122 patients with cSCC, aged 42.7 to 97 years at first diagnosis; of these, 58 patients with LSCC and 64 with ESCC, and 89.3% were male. We recorded a mean follow-up time of 26.1 months.

In accordance with our methodology regarding study design, the matched pairs were constituted based on the occurrence of a nodal disease. The distribution in the metastasis vs. non-metastasis group was as follows: the selected subjects with LN0 (no nodal disease, n = 61) were compared to LN+ subjects (nodal manifestation, n = 61). Nodal disease occurred in 53.8% of female and 49.5% of male patients.

In the Lip-LN0 group, the mean age was 73.77 years and the mean tumor depth 7.27mm; in the Lip-LN+ group, the mean age was 70.58 years and the mean tumor depth 6.88mm. The mean age was higher in the Ear-LN0 (79.92 years) and Ear-LN+ groups (78.35 years), which had mean tumor depths of 7.70mm and 9.14mm, respectively. All patients had an R0 tumor clearance.

### 3.2 Clinical pathology in the study population

**Figure 2:**
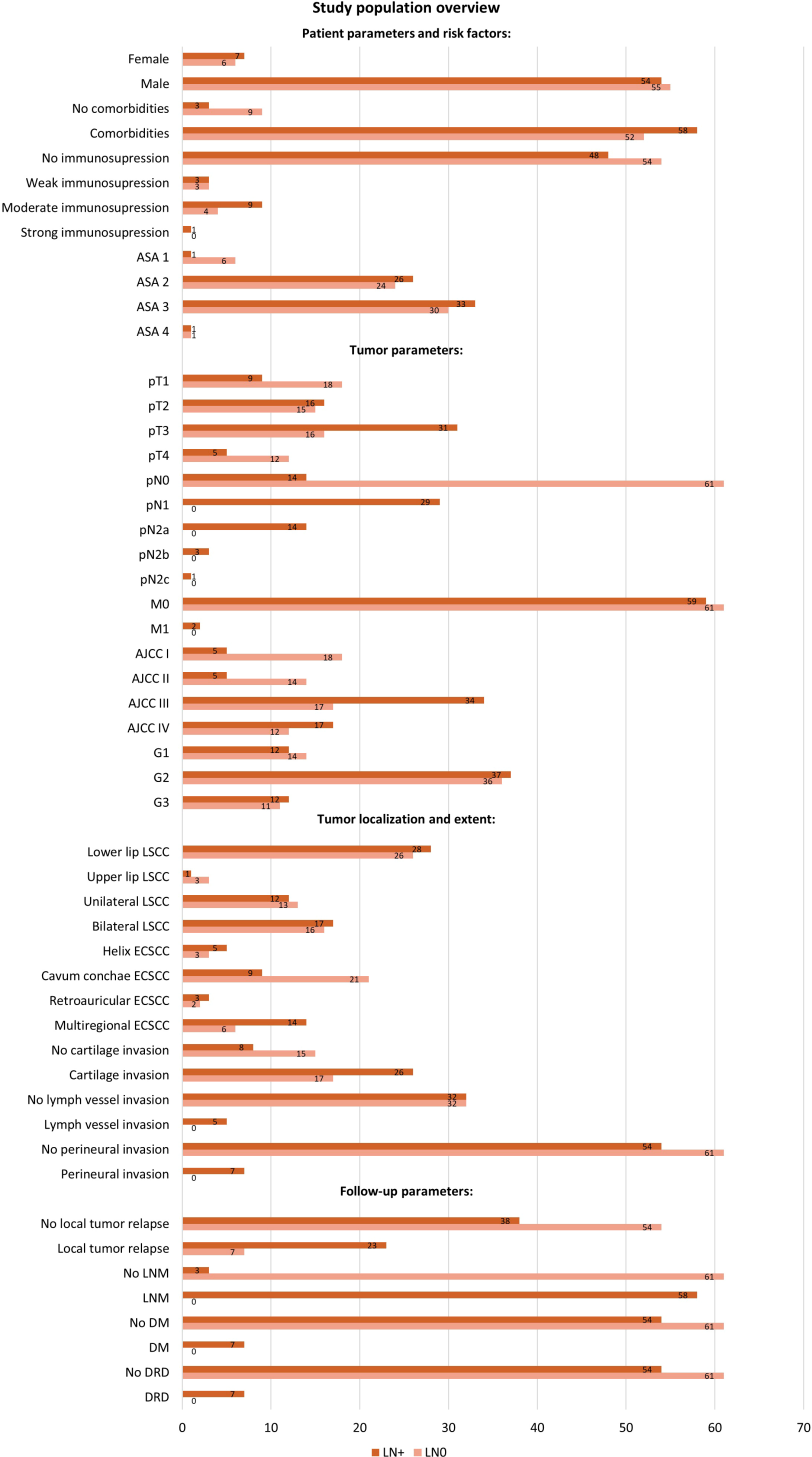
Study population overview with distribution of patients by variables in the LN0 and LN+ groups. The diagram shows the number of cases registered, categorized by patient parameters and risk factors (gender, comorbidities, immunosuppression, ASA Classification), tumor parameters (TNM-stage, AJCC-stage, tumor grading), tumor localization, tumor invasion variables, and follow-up parameters (local relapse, LNM, DM, DRD). Patients without initial nodal disease (pN0) but who developed LNM at follow-up were included in the LN+ group. ASA, American Society of Anesthesiologists; AJCC, American Joint Committee on Cancer; DM, distant metastasis; DRD, disease related death; LN0, no nodal disease; LN+, positive lymph node metastasis; LNM, lymph node metastasis.

This section of the results presents the incidence of LNM in relation to various recorded clinical parameters in our study population. **Figure 2** offers a detailed overview of the study population.

The majority of patients (90.2%) presented comorbidities. Among patients without comorbidities, only 25% developed an LNM, whereas 52.7% of patients with co-occurring diseases exhibited LNM. However, the distribution of subjects with comorbidities in LN+ and LN0 group was similar and not significantly different (52.7% LN+, 47.3% LN0, Chi-Square test, p = 0.068).

Regarding the lip tumor localization, 25.0% of the cases with upper lip tumors developed an LNM, compared to 51.9% of the cases with a lower lip tumor (Fisher’s exact test, p = 0.611). Patients with unilateral tumor expansion developed an LNM in 48.0% of cases. Similarly, 51.5% of those with bilateral tumor expansion developed an LNM (Chi-Square-Test, p = 0.791).

The localization of the external ESCC tumor was statistically significant. An LNM occurred in 62.5% of the subjects with a tumor on the helix auricularis; 30.0% in subjects with tumor of the cavum conchae; 60.0% of those on the retroauricular/posterior side; and 70% of subjects with tumors extending to more than one region (Likelihood ratio, p = 0.030). The extent of the ESCC was significant for metastatic development: LNM occurred in 32.4% of tumors limited to one auricular subsite, 65% of tumors extending to multiple regions, and 100% of the tumors with infiltration of surroundings beyond the auricle (Likelihood ratio, p = 0.001).

Tumor grading was not significantly associated with the presence of LNM. We recorded an LNM in 46.2% of cases of well differentiated tumors (G1), in 50.07% of moderately differentiated tumors (G2) and in 52.2% of poorly differentiated tumors (G3) (Chi-Square-Test, p = 0.900). We have observed LNM in cases with perineural tumor invasion or lymph vessel invasion (perineural invasion, Fisher’s exact test, p = 0.013; lymph vessel invasion, Fisher’s exact test, p = 0.057). Considering the cases with tumor invasion, 60.5% of the patients with cartilage invasion also had an LNM, in comparison to 34.8% of cases with no cartilage invasion (Chi-Square-Test, p = 0.047). Patients without LNM developed local relapse during follow-up in 23.3% of cases, in contrast to 37.7% of cases with LNM. (Chi-Square-Test, p = 0.001). Of 115 patients without DM, only 47% had an LNM, and the other seven patients with DM all developed an LNM (Fisher’s exact test, p = 0.013). All cases with DM were also recorded as a DRD.

### 3.3 Distribution of EZH2 expression score in the study population

**Figure 3:**
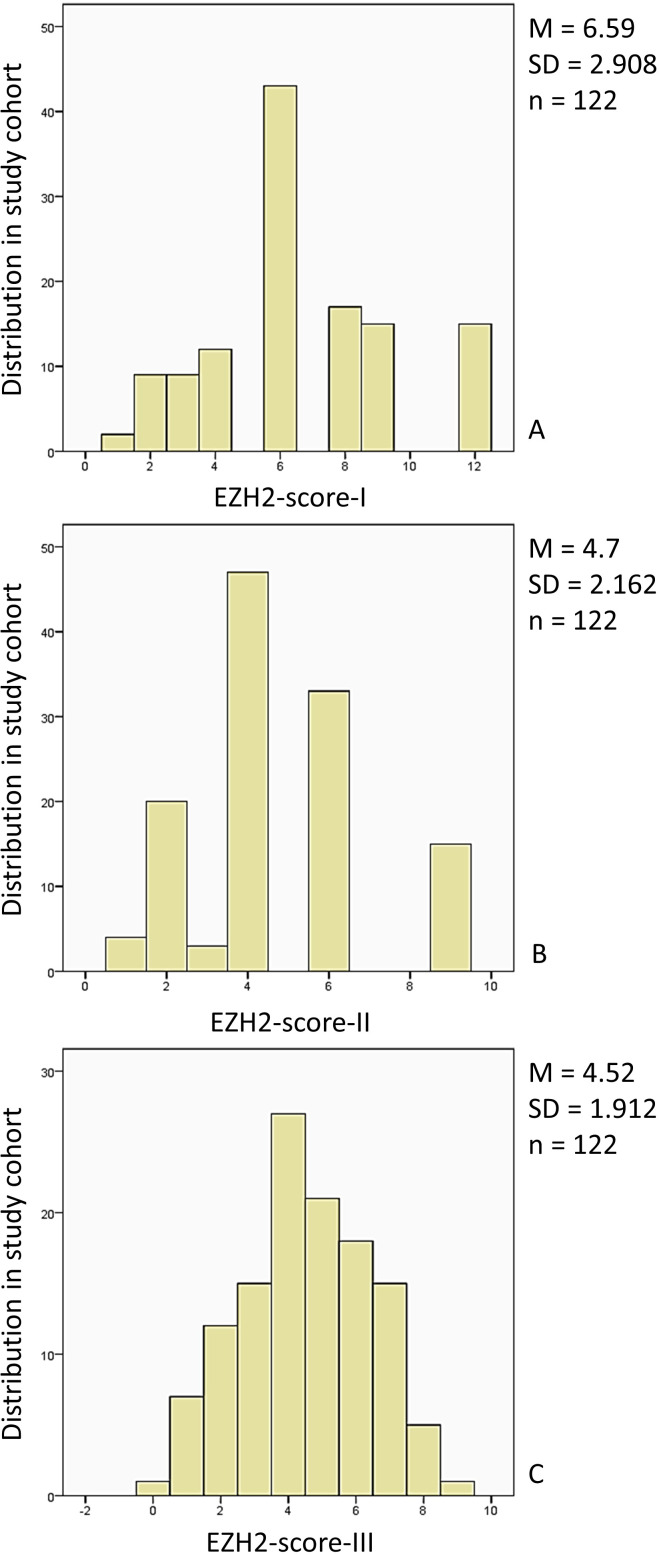
Distribution of the study population in the EZH2-score categories: **(A)** The study cohort (n = 122, 100%) was distributed within the EZH2-score-I category (0–12) (0): n = 0, 0% (1), n = 2, 1.6% (2), n = 9, 7.4% (3), n = 9, 7.4%, (4) n = 12, 9.8%, (5) n = 0, 0%, (6) n = 43, 35.2%, (7) n = 0, 0%, (8) n = 17, 13.9%, (9) n = 15, 12.3%, (10) n = 0, 0%, (11) n = 0, 0%, (12) n = 15, 12.3%. **(B)** The study cohort (n = 122, 100%) distribution for the EZH2-score-II (0–9) was: (0) n = 0, 0%, (1) n = 4, 3.3%, (2) n = 20, 16.4%, (3) n = 3, 2.5%, (4) n = 47, 38.5%, (5) n = 0, 0%, (6) n = 33, 27%, (7) n = 0, 0%, (8) n = 0, 0%, (9) n = 15, 12.3%. **(C)** The study cohort (n = 122, 100%) EZH2-score-III (0–10) distribution was: (0) n = 1, 0.8%, (1) n = 7, 5.7%, (2) n = 12, 9.8%, (3) n = 15, 12.3%, (4) n = 27, 22.1%, (5) n = 21, 17.2%, (6) n = 18, 14.8%, (7) n = 15, 12.3%, (8) n = 5, 4.1%, (9) n = 1, 0.8%, (10) n = 0, 0%. M, mean; SD, standard deviation; n, study population.

The first part of the results offers a detailed overview of the study population, its distribution in the LN+ and LN0 groups, and correspondence with LNM. We present the distribution of the study cohort within the three EZH2-score categories in **Figure 3**. Finally, we will give an overview of the analyzed EZH2-scores in correlation with clinical outcome parameters.

### 3.4 EZH2-score-I – III: a high EZH2 expression score corresponded with both poor and positive outcome parameters

Given the demonstrated potential of EZH2 expression analysis, there is an essential demand for a standardized method for expression analysis. As previously outlined in the methodology, our objective was to assess the clinical suitability of different scores, taking into account both the percentage of stained cells and the intensity, while evaluating relevant clinical parameters. All three versions of the calculated EZH2-score were analyzed according to LN0/LN+, DRD, disease progression, DM, local relapse, pT-classification, AJCC-stage, and grading.

**Figure 4:**
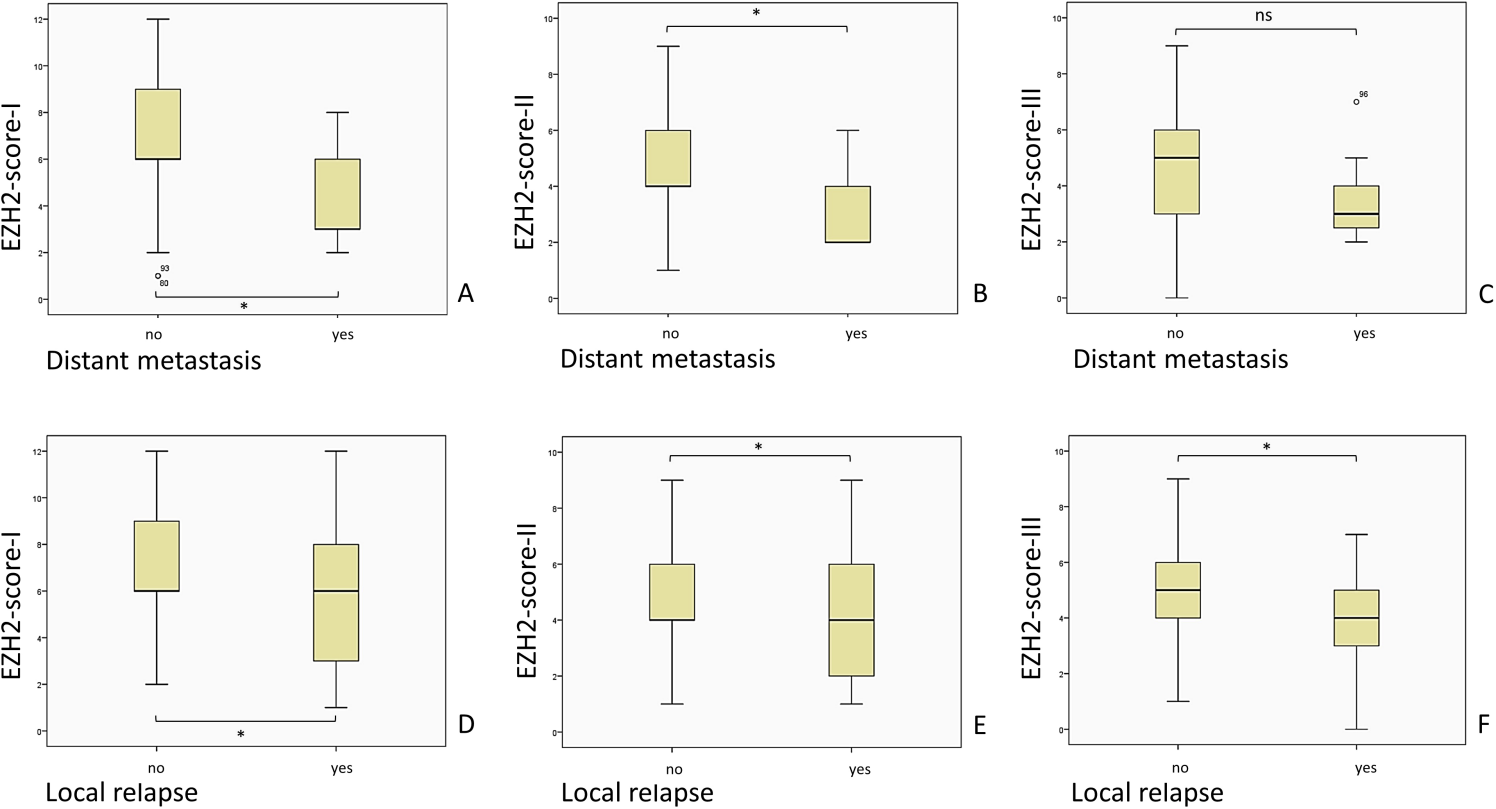
The mean EZH2-score was significantly lower when DM or local relapse was registered. The total of patients included was n = 122. The Panels **(A-F)** display the EZH2-scores for the aforementioned analysis and the distribution by clinical parameter was as follows: DM (no) n = 115, DM (yes) n = 7; Local relapse (no) n = 93, Local relapse (yes) n = 29; In regard to the statistical outcome, the following data is available: **(A)** p = 0.038, Mann-Whitney-U-Test; **(B)** p = 0.039, Mann-Whitney-U-Test; **(C)** p = 0.131, Mann-Whitney-U-Test; **(D)** p = 0.025, Mann-Whitney-U-Test; **(E)** p = 0.027, Mann-Whitney-U-Test; **(F)** p = 0.038, Mann-Whitney-U-Test; ns = p > 0.005; * = p ≤ 0.05.

**Figure 5:**
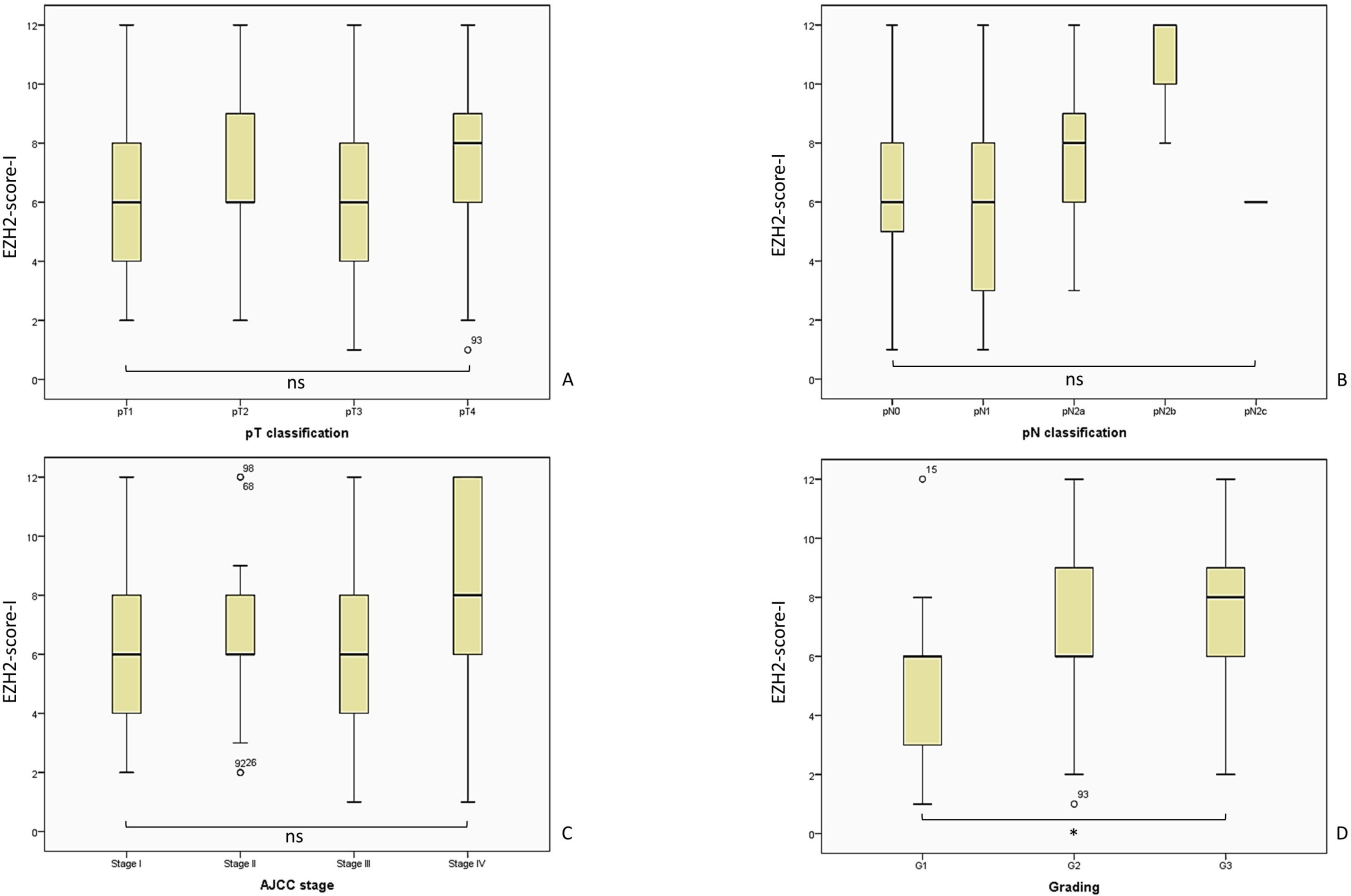
Overview of the analyzed EZH2-score-I and the corresponding tumor parameters. We included n = 122 patients for the analysis of the EZH2-scores I - III, while the distribution was for pT1 n = 27, pT2 n = 31, pT3 n = 47, pT4 n = 17; pN0 = 75, pN1 = 29, pN2a = 14, pN2b = 3, pN2c = 1; AJCC I n = 23, AJCC II n = 19, AJCC III n = 51, AJCC IV n = 29; G1 n = 26, G2 n= 73, G3 n = 23. The statistical outcome was determined for **(A)** EZH2-score-I and pT-classification, Kruskal-Wallis-Test, p = 0.337; **(B)** EZH2-score-I and pN-classification, Kruskal-Wallis-Test, p = 0.068; **(C)** EZH2-score-I and AJCC-stage, Likelihood ratio, p = 0.569; **(D)** EZH2-score-I and tumor grading, Likelihood ratio, p = 0.061. AJCC, American Joint Committee on Cancer; ns = p > 0.005; * = p ≤ 0.05.

**Figure 6:**
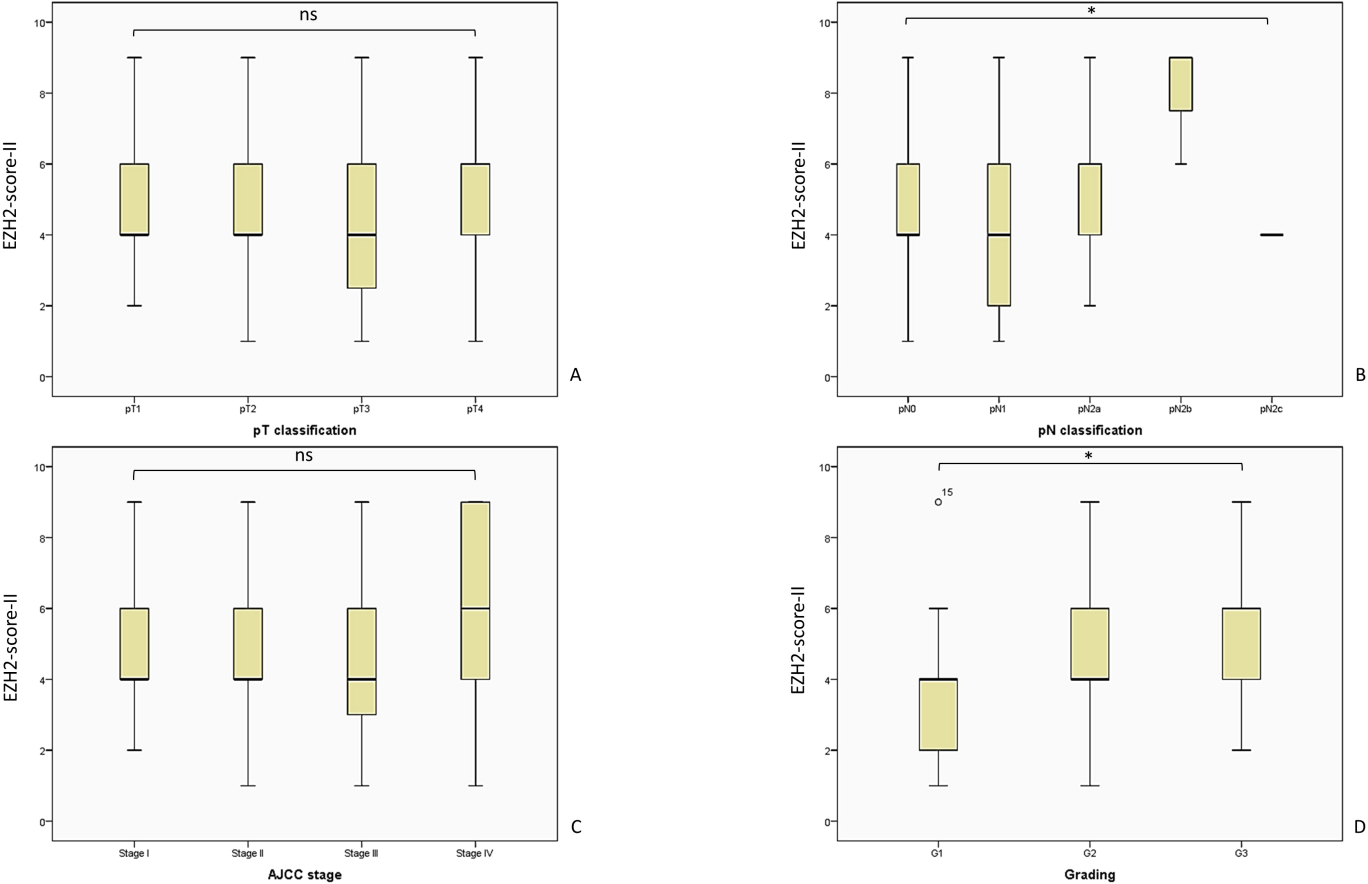
Here we present the analyzed EZH2-score-II and the evaluated tumor parameters. The patient cohort is consistent with the analysis of EZH2-score I and III, as described in **Figure 5**. **(A)** EZH2-score-II and pT-classification, Kruskal-Wallis-Test, p = 0.459; **(B)** EZH2-score-II and pN-classification, Kruskal-Wallis-Test, p = 0.038; **(C)** EZH2-score-II and AJCC-stage, Likelihood ratio, p = 0.301; **(D)** EZH2-score-II and tumor grading, Likelihood ratio, p = 0.013. AJCC, American Joint Committee on Cancer; ns = p > 0.005; * = p ≤ 0.05.

**Figure 7:**
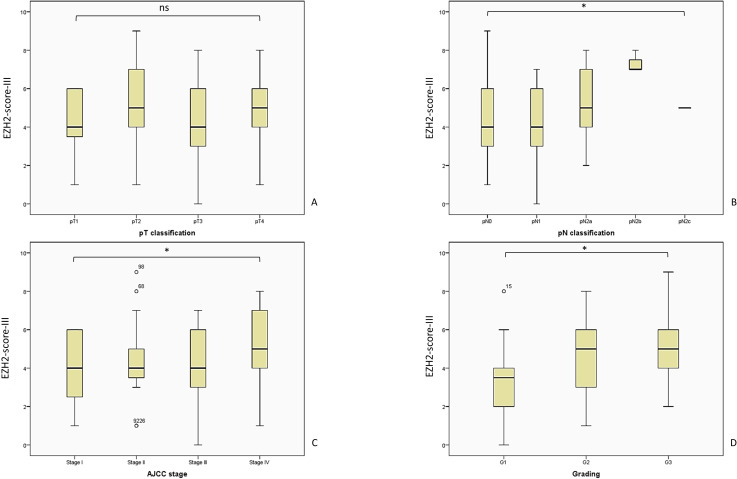
The analysis of the EZH2-score-III showed a consistent outcome and corresponding with a poor outcome regarding tumor parameters. The patient distribution remains consistent with the previously described pattern, as illustrated in **Figure 5**. **(A)** EZH2-score-III and pT-classification, Kruskal-Wallis-Test, p = 0.342; **(B)** EZH2-score-III and pN-classification, Kruskal-Wallis-Test, p = 0.036; **(C)** EZH2-score-III and AJCC-stage, Likelihood ratio, p = 0.08; **(D)** EZH2-score-III and tumor grading, Likelihood ratio, p = 0.041. AJCC, American Joint Committee on Cancer; ns = p > 0.005; * = p ≤ 0.05.

In the following part we present the evaluated mean (M) EZH2-scores in correlation with the clinical outcome parameters, along with the standard deviation (SD), confidence interval (CI), the applied statistical test and p-value. **Figure 4** provides an overview of the evaluated EZH2-scores and clinical outcome parameters, while **Figures 5**–**7** displays the evaluated EZH2-scores and tumor outcome parameters.

#### 3.4.1 EZH2-score-I: a lower score correlated with occurrence of DM and local relapse, while a higher EZH2-score corresponded with high grading

The overall analysis of the EZH2-score in the entire cohort (comprising both LSCC and ESCC) showed no significant difference regarding the occurrence of nodal disease. The mean EZH2-score-I in the LN0 group was 6.79 (SD = 2.74; 95% CI 6.08–7.49), while the LN+ group had a mean of 6.39 (SD = 3.07; 95% CI 5.61–7.18); Likelihood ratio, p = 0.130; Mann-Whitney-U-Test, p = 0.399.

It is of significant clinical importance to examine the EZH2-score-I within the Lip-LN+, Lip-LN0, Ear-LN+, and Ear-LN0 subgroups. The Lip-LN0 cohort had a mean score of 7.31 (SD = 2.71; 95% CI 6.28–8.34), while the Lip-LN+ group had a mean score of 6.62 (SD = 2.55 CI 95% 5.65–7.59); t-test, p = 0.323. Furthermore, not statistically significant, the Ear-LN0 group had a mean of 6.31 (SD = 2.72; 95% CI 5.33–7.30), while the Ear-LN+ group had a mean score of 6.19 (SD = 3.50; 95% CI 4.92–7.45); t-test, p = 0.874.

We followed evaluation of the correlation between the EZH2-score-I and clinical outcome parameters, commencing with the occurrence of DRD. The mean score of patients without DRD (M = 6.64; SD = 2.75 95% CI 6.11–7.17) was higher compared to patients with DRD (M = 6.25; SD = 3.85, 95% CI 4.20–8.30); Mann-Whitney-U-Test, p = 0.610; Likelihood ratio, p = 0.096.

Patients without disease progression had a higher mean EZH2-score (M = 6.95; SD = 2.75; 95% CI 6.27–7.64) than patients with disease progression (M = 6.18; SD = 3.04; 95% CI 5.37–6.98), however no statistical significance was demonstrated; Mann-Whitney-U-Test, p = 0.103; Likelihood ratio, p = 0.063.

Interestingly, patients with DM had a significantly lower EZH2-score-I (M = 4.43; SD = 2.22; 95% CI 2.37–6.49) than patients without DM (M = 6.72; SD = 2.90; 95% CI 2.19–7.26); Mann-Whitney-U-Test, p = 0.038; Likelihood ratio, p = 0.098. It is important to note that the number of patients with DM is relatively low, which consequently affects the potential impact of the results.

In addition, the mean EZH2-score-I of patients with local relapse (M = 5.52; SD = 2.95; 95% CI 4.39–6.64) was lower (Mann-Whitney-U-Test, p = 0.025; Likelihood ratio, p = 0.174) than in patients without a local relapse (M = 6.92; SD = 2.82; 95% CI 6.34–7.51).

The correlation analysis between the EZH2-score-I and tumor parameters pT-, pN-classification, AJCC stage and tumor grading is presented in **Figure 5**. A similar presentation of the results has been conducted for EZH2-score-II and -III.

The pT-classification did not correlate with the EZH2-score-I: pT1 M = 6.11; SD = 2.35; 95% CI 5.18–7.04; pT2 M = 7.10; SD = 2.87; 95% CI 6.04–8.15; pT3 M = 6.28; SD = 3.09; 95% CI 5.37–7.18; and pT4 M = 7.29; SD = 3.19; 95% CI 5.65–8.94; Kruskal-Wallis-Test, p = 0.337.

A similar outcome was observed regarding the pN-classification, which was not associated with the EZH2-score-I: pN0 M = 6.49; SD = 2.74; 95% CI 5.86–7.13; pN1 M = 5.90; SD = 3.02; 95% CI 4.74–7.05; pN2a M = 7.71; SD = 2.97; 95% CI 6.00–9.43; and pN2b M = 10.67; SD = 2.30; 95% CI 4.93–16.40 (Kruskal-Wallis-Test, p = 0.068).

The EZH2-score-I showed a trend of increasing with AJCC stage, but the discrepancy in the score was not statistically significant: AJCC-I M = 6.13; SD = 2.56; 95% CI 5.02–7.24; AJCC-II M = 6.47; SD = 2.77; 95% CI 5.14–7.81; AJCC-III M = 6.16; SD = 2.71; 95% CI 5.39–6.92; AJCC-IV M = 7.79; SD = 3.35; 95% CI 6.52–9.07; Kruskal-Wallis-Test, p = 0.118; Likelihood ratio, p = 0.569.

Lastly, the analysis of the tumor grading revealed a surprising correlation. A high EZH2-score-I corresponded statistically with a higher tumor-grading: G1 M = 4.85; SD = 2.34; 95% CI 3.90–5.79; G2 M = 7.00; SD = 2.98; 95% CI 6.30–7.70; G3 M = 7.26; SD = 2.54; 95% CI 6.16–8.36 (Kruskal-Wallis-test, p = 0.001; Likelihood ratio, p = 0.061; crosstab analysis linear-by-linear association, p = 0.003).

#### 3.4.2 EZH2-score-II: a lower score corresponded with the presence of DM and local relapse, while a higher score was linked to a higher pN- and grading status

As was done previously, we conducted an evaluation of the EZH2-score-II in the total collective (both LSCC and ESCC), regarding the occurrence of LNM. The mean EZH2-score-II in the LN0 group was 4.84 (SD = 2.02; 95% CI 4.32–5.36), while the LN+ group has a mean of 4.57 (SD = 2.29; 95% CI 3.99–5.16); Likelihood ratio, p = 0.690; Mann-Whitney-U-Test, p = 0.386. This resulted in no statistically significant outcome.

We proceeded to undertake a comparative analysis of the EZH2-score-II between the subgroups Lip-LN+, Lip-LN0, Ear-LN+, and Ear-LN0, however the difference was not significant (t-test, p = 0.873). The Lip-LN0 subgroup had a mean score of 5.17 (SD = 2.00; 95% CI 4.41–5.93), while the Lip-LN+ group had a mean score of 4.72 (SD = 1.92 CI 95% 3.99–5.46); t-test p = 0.388. The Ear-LN0 group had a mean of 4.53 (SD = 2.03; 95% CI 3.80–5.26), while the Ear-LN+ group had a mean score of 4.44 (SD = 2.61; 95% CI 3.49–5.38).

Furthermore, the EZH2-score-II of patients without a DRD (M = 4.73; SD = 2.05 95% CI 4.33–5.12) was not statistically different to those with DRD (M = 4.56; SD = 2.82, 95% CI 3.06–6.07); Mann-Whitney-U-Test, p = 0.640; Likelihood ratio, p = 0.183.

Although disease progression represents a clinical concern, the correlation with EZH2-score-II was not statistically significant. Patients without disease progression had a higher mean EZH2-score-II (M = 4.95; SD = 2.07; 95% CI 4.44–5.47) than patients with disease progression (M = 4.42; SD = 2.24; 95% CI 3.83–5.02); Mann-Whitney-U-Test, p = 0.099; Likelihood ratio, p = 0.430.

As with the findings of the EZH2-score-I, patients with DM had a significantly lower EZH2-score-II (M = 3.14; SD = 1.57; 95% CI 1.69–4.60) than patients without DM (M = 4.80; SD = 2.16; 95% CI 4.40–5.20); Mann-Whitney-U-Test, p = 0.039; Likelihood ratio, p = 0.152.

The incidence of local relapse represents a significant clinical interest. The mean EZH2-score-II of patients with a local relapse (M = 3.97; SD = 2.12; 95% CI 3.16–4.78) was significant lower (Mann-Whitney-U-Test, p = 0.027; Likelihood ratio, p = 0.096) than patients without local relapse (M = 4.94; SD = 2.13; 95% CI 4.50–5.37).

The analysis of the score and various tumor marker yielded intriguing results, although the pT-classification did not correlate with the mean EZH2-score-II: pT1 M = 4.41; SD = 1.67; 95% CI 3.75–5.07; pT2 M = 5.03; SD = 2.19; 95% CI 4.23–5.84; pT3 M = 4.49; SD = 2.30; 95% CI 3.81–5.17; pT4 M = 5.18; SD = 2.40; 95% CI 3.94–6.41; Kruskal-Wallis-Test, p = 0.459. The correlation analysis along with the other tumor parameters is presented in **Figure 6**.

Interestingly, a higher EZH2-score-II was associated with a higher pN-status (overall cohort = LN0 + LN+): pN0 M = 4.65; SD = 2.01; 95% CI 4.19–5.12; pN1 M = 4.14; SD = 2.21; 95% CI 3.30–4.98; pN2a M = 5.50; SD = 2.34; 95% CI 4.15–6.85; pN2b M = 8.00; SD = 1.73; 95% CI 3.70–12.30 (Kruskal-Wallis-Test, p = 0.038).

In the context of the AJCC-staging system the EZH2-score-II did not result in significant discrepancy: AJCC-I M = 4.48; SD = 1.80; 95% CI 3.70-5.26; AJCC-II M = 4.587; SD = 2.06; 95% CI 3.58-5.57; AJCC-III M = 4.37; SD = 2.01; 95% CI 3.81-4.94; AJCC-IV M = 5.55; SD = 2.58; 95% CI 4.57-6.54; Kruskal-Wallis-Test, p = 0.203; Likelihood ratio, p = 0.301.

In alignment with prior outcomes and of considerable clinical interest, a high EZH2-score-II was associated with a higher tumor-grading: G1 M = 3.46; SD = 1.65; 95% CI 2.79–4.13; G2 M = 5.03; SD = 2.25; 95% CI 4.50–5.55; G3 M = 5.09; SD = 1.90; 95% CI 4.26–5.91 (Kruskal-Wallis-test, p = 0.002; Likelihood ratio, p = 0.013; crosstab analysis linear-by-linear association, p = 0.007).

#### 3.4.3 EZH2-score-III: a lower score correlated with occurrence of local relapse, a higher score was linked to higher pN-, AJCC-, and grading classification

The evaluation of the final score model was conducted in accordance with the same procedure as the initial evaluations. The analysis commenced with an assessment of the score in the overall collective (encompassing both LSCC and ESCC) regarding the presence of nodal disease. This resulted in no notable distinction. The mean EZH2-score-III in the LN0 group was 4.61 (SD = 1.92; 95% CI 4.11–5.10), while the LN+ group had a mean of 4.43 (SD = 1.91; 95% CI 3.94–4.92); Likelihood ratio, p = 0.833; Mann-Whitney-U-Test, p = 0.624.

We proceeded with the analysis of the EZH2-score-III in the LN+ and LN0 subgroups of LSCC and ESCC, observing no statistically significant results. The mean score of the Lip-LN0 subgroup was 4.93 (SD = 1.83; 95% CI 4.23–5.63), while the Lip-LN+ group had a mean score of 4.69 (SD = 1.51 CI 95% 4.11–5.27); t-test, p = 0.586. Lacking statical difference, the Ear-LN0 group had a mean of 4.31 (SD = 1.99; 95% CI 3.59–5.03), while the Ear-LN+ group had a mean score of 4.19 (SD = 2.20; 95% CI 3.39–4.98); t-test, p = 0.813.

The mean score of patients without DRD (M = 4.57; SD = 1.80 95% CI 4.22–4.91) was also not statistically different to that of those with DRD (M = 4.19; SD = 2.56, 95% CI 2.82–5.55); Mann-Whitney-U-Test, p = 0.676; Likelihood ratio, p = 0.167.

Patients without disease progression had a higher mean EZH2-score (M = 4.69; SD = 1.96; 95% CI 4.21–5.18) than patients with disease progression (M = 4.32; SD = 1.85; 95% CI 3.82–4.81); Mann-Whitney-U-Test, p = 0.288; Likelihood ratio, p = 0.556.

The cohort with DM had a lower mean EZH2-score-III (M = 3.57; SD = 1.81; 95% CI 1.90–5.25) than patients without DM (M = 4.57; SD = 1.91; 95% CI 4.22–4.93); Mann-Whitney-U-Test, p = 0.131; Likelihood ratio, p = 0.192. In contrast with previous results concerning EZH2-score-I and EZH2-score-II, the result was not statistically relevant.

Similar to the first two EZH2-scores, patients with local relapse had a lower mean score (M = 3.86; SD = 1.95; 95% CI 3.12–4.61) than patients without local relapse (M = 4.72; SD = 1.86; 95% CI 4.34–5.10); Mann-Whitney-U-Test, p = 0.038; Likelihood ratio, p = 0.091.

As with the other scores, an overview of the EZH2-score-III and the tumor parameters is shown in **Figure 7**. In light of the observation that a high EZH2-score-I and -II correlated with tumor variables, we postulated that a comparable situation might be at play with regard to the final score model. However, the analysis of the pT-classification did not correlate with the EZH2-score-III: pT1 M = 4.30; SD = 1.61; 95% CI 3.66–4.93; pT2 M = 4.97; SD = 1.99; 95% CI 4.24–5.70; pT3 M = 4.21; SD = 1.95; 95% CI 3.64–4.79; and pT4 M = 4.88; SD = 2.02; 95% CI 3.84–5.92; Kruskal-Wallis-Test, p = 0.342.

In contrast, the remaining tumor variables demonstrate a consistent outcome. A high EZH2-score-III showed a significant correlation with increasing pN status, over both groups (LN0 + LN+): pN0 M = 4.45; SD = 1.89; 95% CI 4.02–4.89; pN1 M = 4.03; SD = 1.89; 95% CI 3.31–4.76; pN2a M = 5.21; SD = 1.71; 95% CI 4.22–6,21; pN2b M = 7.33; SD = 0.57; 95% CI 5.90–8.77 (Kruskal-Wallis-Test, p = 0.036).

As the sole score model to demonstrate a correlation with the AJCC-stage, the EZH2-score-III showed a significant increase with increasing AJCC-stage: AJCC-I M = 4.22; SD = 1.70; 95% CI 3.48–4.95; AJCC-II M = 4.58; SD = 2.09; 95% CI 3.57–5.59; AJCC-III M = 4.25; SD = 1.84; 95% CI 3.74–4.77; AJCC-IV M = 5.17; SD = 2.00; 95% CI 4.41–5.93; Kruskal-Wallis-Test, p = 0.205; Likelihood ratio, p = 0.008.

The final parameter analysis revealed a uniform result across all three score models, thereby demonstrating the reliability and consistency of the findings. A high EZH2-score-III was associated with higher tumor-grading: G1 M = 3.31; SD = 1.80; 95% CI 2.58–4.04; G2 M = 4.74; SD = 1.83; 95% CI 4.31–5.17; G3 M = 5.15; SD = 1.74; 95% CI 4.42–5.93 (Kruskal-Wallis-test, p = 0.001; Likelihood ratio, p = 0.041; crosstab analysis linear-by-linear association, p = 0.001).

### 3.5 EZH2 extent and intensity did not correspond with the occurrence of LNM

**Figure 8:**
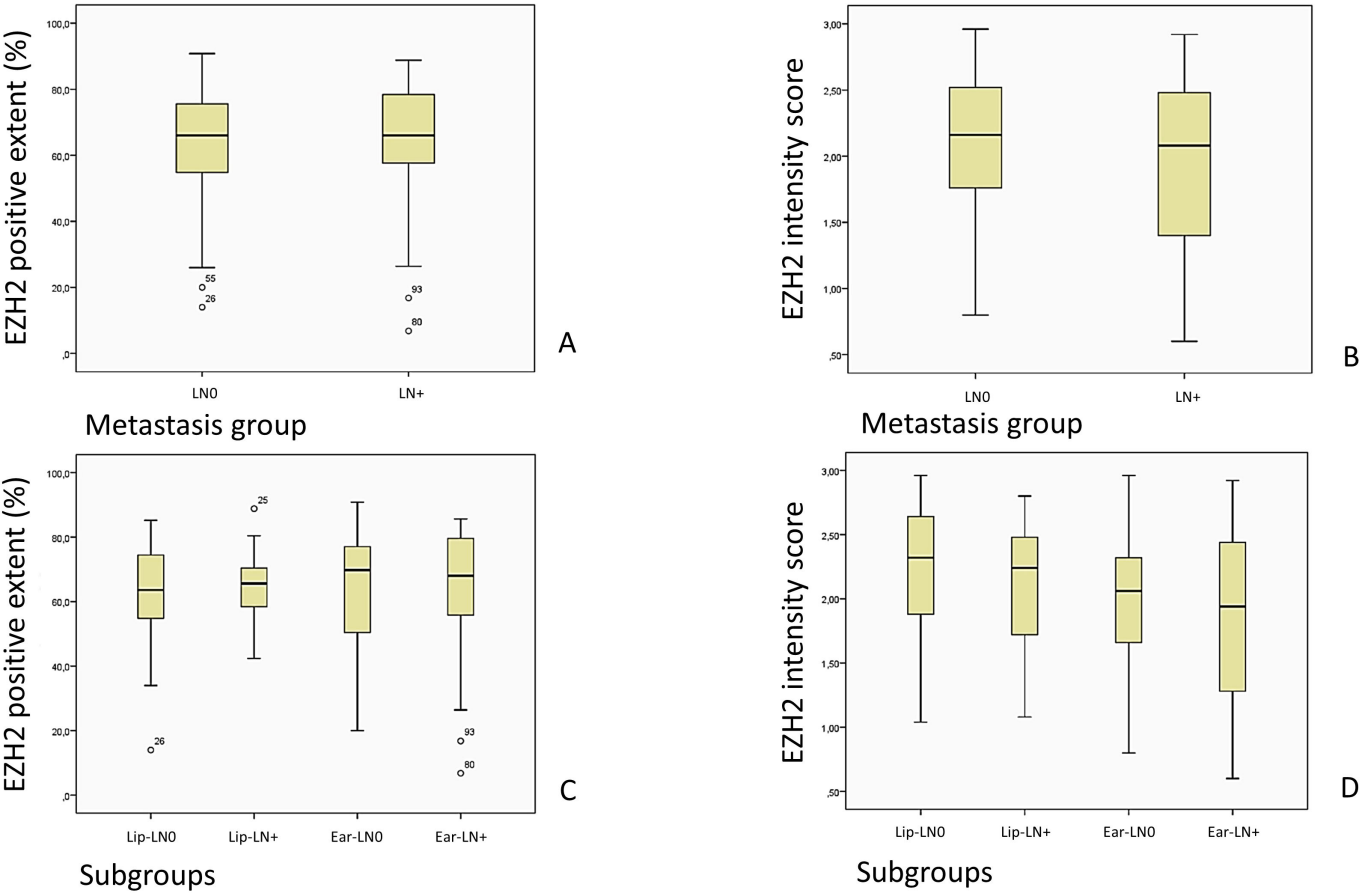
Images **(A, C)** show an overview of the EZH2-stained cells described as EZH2 positive extent (%) in the LN0 and LN+ group, while **(B, D)** show the overview of the EZH2 staining intensity, corresponding to the EZH2 intensity score (0–3), in the LN0 and LN+ group.

**Figure 9:**
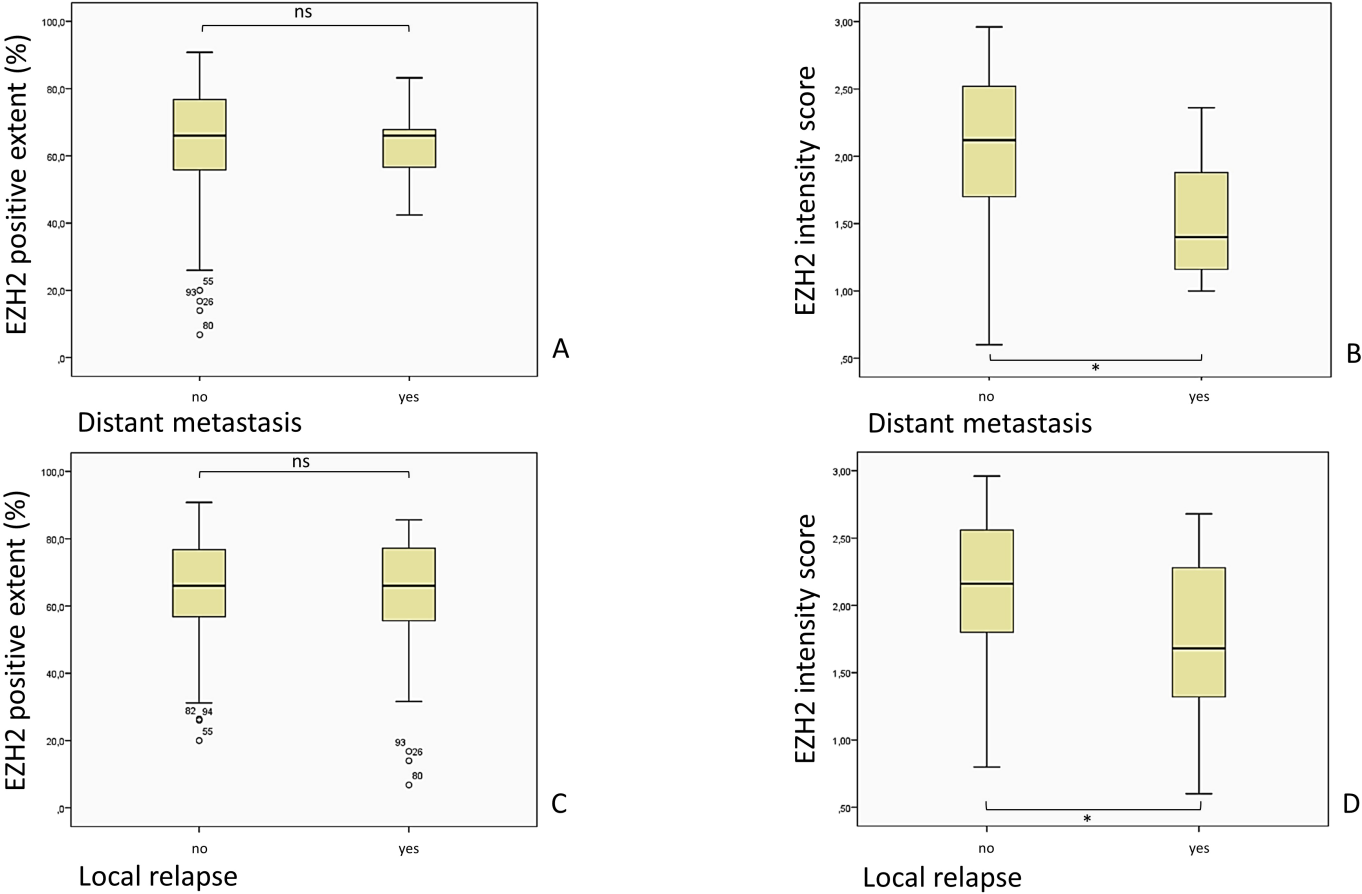
Comparison of the EZH2-stained cells (EZH2 positive extent %) and the intensity of the EZH2 staining, corresponding to the EZH2 intensity score (0– 3), in patients with and without DM and local relapse of the tumor. **(A)** p = 0.648, Mann-Whitney-U-Test; **(B)** p = 0.030, Mann-Whitney-U-Test; **(C)** p = 0.528, Mann-Whitney-U-Test; **(D)** p = 0.004, Mann-Whitney-U-Test).

The extent of EZH2-positive stained cells did not correlate statistically with any evaluated parameters. An overview of the EZH2-staining and its intensity in the evaluated groups is shown in **Figure 8** and its correlation to DM and local relapse in **Figure 9**.

The matched pairs approach adopted in this study precluded the possibility of a control group comparison. Instead, the analysis focused on the examination of tumor tissue samples. While the comparisons of the amount of stained cells within subgroups lacked statistical significance, the majority of tissue examined exhibited a staining over 50%. This suggests that a high level of EZH2 expression is widely detectable in tumor tissue. The distribution of the EZH2-positive stained cells (n = 100%) in the subgroups was as follows: Lip-LN0 1. <25% n = 3.4% 2. 25–50% n = 10.3% 3. 50–75% n = 62.1% 4. >75% n = 24.1%; Lip-LN+ 1. <25% n = 0% 2. 25–50% n = 10.3% 3. 50–75% n = 69.0% 4. >75% n = 20.7%; Ear-LN0 1. <25% n = 3.1% 2. 25–50% n = 21.9% 3. 50–75% n = 43.8% 4. >75% n = 31.3%; Ear-LN+1. <25% n = 6.3% 2. 25–50% n = 12.5% 3. 50–75% n = 43.8% 4. >75% n = 37.5%; Likelihood ratio, p = 0.435.

The mean (%) of EZH2 positive stained cells in the LN0 group was 63.13 (SD = 17.35 CI 58.69–67.57), while the LN+ group had a mean of 64.19 (SD = 16.69, CI 59.92–68.47, Mann-Whitney-U-Test, p = 0.691).

A similar outcome followed for the comparison of the EZH2 intensity score, which did not diverge significantly in the LN0 group (M = 2.09; SD = 0.53; 95% CI 1.95–2.22) from the LN+ group (M = 1.97; SD = 0.59; 95% CI 1.81–2.12), Mann-Whitney-U-Test, p = 0.292.

### 3.6 EZH2 staining intensity correlates with the risk of DM and local relapse

A noteworthy outcome has emerged from the examination of EZH2 intensity in conjunction with clinical parameters. In alignment with the findings of the preceding EZH2-score analysis, a diminished intensity was associated with superior outcomes in terms of occurrence of distant metastasis and local relapse. Staining intensity corresponded significantly with the occurrence of DM (Mann-Whitney-U-Test, p = 0.030): the mean EZH2 intensity score of patients with DM (M = 1.54; SD = 0.54; 95% CI 1.04–2.05) was lower than in patients without DM (M = 2.06; SD = 0.56; 95% CI 1.95–2.16). As previously stated for the EZH2-scores, the same limitation regarding the small sample size of patients with DM is applicable here as well.

Additionally, the mean intensity score of subjects with a local relapse (M = 1.75; SD = 0.57; 95% CI 1.53–1.97) was significant lower (Mann-Whitney-U-Test, p = 0.004) than the mean in subjects without a local relapse (M = 2.11; SD = 0.54; 95% CI 2.00–2.22).

## 4 Discussion

### 4.1 Strengths and limitations of the study design

We used a matched pair approach for this study, with the advantage of matching the subjects by multiple variables and eliminating their effect on EZH2 expression, the examined variable. EZH2 expression was not only assessed by the extent of immunohistochemical stained cells, but also by staining intensity and a combination of both, allowing evaluation of further potential correlations. Matching was possible due to the high number of rare cases with high-risk SCCs, but also implies a selection bias by allocation of the subjects to the LN0 and LN+ groups. It is also important to mention the small sample sizes of some subgroups, which makes it difficult to draw definite statistical conclusions. The comorbidities were of disparate nature overall and also influenced the overall survival. Due to the heterogeneity of the comorbidities, it was not feasible to match them with precision, which may result in distortions. Typically, a retrospective study is more liable to sampling bias, lower data quality, and confounding by indication, the latter being reduced in our study by our inclusion criteria.

Furthermore, allocation of the staining intensity was not automated, leading to potential observer bias. However, we underline the accordant analysis of EZH2-expression between the investigators as a strength of the study, implying a consistent examination of the slides. Nonetheless, using a score to interpret the expression is a potential debatable subject and therefore analysis and outcomes may vary.

In addition, the source of the LSCC (oral mucosa/white or red epithelia of the lip) is not distinguishable, but of high importance regarding clinical classification, aggressiveness, and outcome (14). We included both upper and lower lip SCCs, due to their similar prognostic outcomes (6, 34). The limitations of our study design have been considered during the semi-quantitative and statistical analyses in order to obtain clinically applicable results.

### 4.2 Heterogenous EZH2 expression in cSCC

Previous data in multiple studies have indicated that high EZH2-expression is linked with oncogenic activity in various cancer entities. However, a poor clinical outcome associated with low EZH2-expression has been likewise reported (20). Despite similar skin anatomy in most body regions, it has been reported that the occurrence and outcome of NMSC differs with its localization (3, 13, 34). Anatomy, embryological development, ultraviolet (UV) exposure, and chronic processes influence tumorigenesis. The auricular, cheek, and lower lip manifestations of SCCs may be due to corresponding fusion planes (35). LSCC and ESCC are therefore often clinically overlapping entities, with different behaviors compared to other SCCs (36, 37). Previous data indicate that EZH2 behavior could be influenced by tumor topography and its histological characteristics (28). A case-control study in Taiwan showed that not all EZH2 genotypes correlate with a higher susceptibility for carcinoma (17). Thus, the cSCC is a heterogenous tumor entity and it is to be expected that LSCC and ESCC do not behave identically to all cutaneous malignancies.

One of the major causes of cSCC in high-risk localization is UV-radiation. Qin et al. (38) reported that UV exposure leads to reduced fibroblast proliferation, enhanced cell apoptosis and fibroblast senescence, reduced the hyaluronic acid level and led to increased mRNA expression of EZH2, while using inoculation of human skin fibroblast cell cultures. Specific inhibition of EZH2 with GlaxoSmithKline126 after UV-radiation resulted in superior fibroblast cell growth and inhibited smad2, smad4 and matrix-metalloproteinase-1 as photoaging molecular pathways by impeding scar repair and deterioration of collagen. It has been discussed that the EZH2-inhibition may reduce H3K9 methylation levels, constraining the arrangement of aging-associated heterochromatic loci.

Furthermore, it has been demonstrated that actinic keratosis and cSCC tissue have significantly increased EZH2 expression in comparison to normal skin: the highest percentage of EZH2 expression has been reported in the early stage of the disease for carcinomas *in situ*, and lower percentages in the invasive carcinoma stage with poor differentiation, possibly due to loss of nuclei and less immunohistochemical staining (39). Our variant development of EZH2-score and tumor grading may be due to different methodical definitions of positive expression or staining. This shows that simple and standardized interpretation of EZH2-expression is important for clinical suitability and comparison, but still has its challenges for demarcating the stages of malignancy. The therapeutic aim of the EZH2 mediated axis in carcinogenesis is complex and reducing it to a dichotomous marker for all skin lesions would be unfounded.

### 4.3 Molecular pathways of EZH2 regulation and future perspectives

The role of the enzyme EZH2 in the physiological context is highly complex and subject to a vast array of regulatory mechanisms. In this study we will only discuss regulatory patterns which serve to illustrate the extensive range of biochemical cascades in which EZH2 exerts influence or is influenced by. It would be advisable to take these mechanisms into account when considering EZH2 modulator treatment.

Interesting results have been concluded regarding the EZH2 and Poly(ADP-ribose) polymerase 1 (PARP1) activity after UV-related DNA damage (40). In physiological conditions the inhibition of PARP1 would lead to upregulation of EZH2 with the functional consequence of an increased H3K27 trimethylation. This would lead to enhanced chromatin compaction and gene silencing. The study verified the hypothesis that DNA damage causes an increased activity of PARP1 resulting in increased Poly(ADP-ribosyl)ation (PARylation) of EZH2. PARylated EZH2 presented reduced methyltransferase activity. Additionally, PARylation of histone H3 was also observed, resulting in loss of affinity of EZH2 to its substrate. The authors validated that UV radiation results mostly in the PARylated isoform of EZH2, which is limited in its enzymatic function. It has been posited, that the inhibition is beneficial in maintaining low EZH2-mediated chromatin condensation at the DNA-damage locus, however the precise DNA-repair mechanism remains unclear. It is uncertain to what extent the elevated EZH2 expression we observed in the tumor tissue is attributable to regulatory compensation mechanisms. However, the inhibition of PARP1 as an additional therapeutic modality in conjunction with EZH2-modulation appears to be a promising approach, and further clinical research on this topic is of interest for clinical therapy integration.

The immunohistochemical method employed in our study, utilizing anti-EZH2 mouse antibodies, resulted in nuclear staining. However, EZH2 expression can be further differentiated into the nuclear soluble and chromatin-bound EZH2. It has been reported that DNA damage results in elevated levels of soluble EZH2 within the nucleus, while the amount of chromatin associated EZH2 is reduced (40). Past literature describes after DNA damage elevated methylated H3K27 levels (41), as well as no significant alterations of the levels of methylated H3K27 (42), but reported radiation and alkylation as different sources of DNA damage. Therefore Caruso et al. (40) suggests various DNA repair regulation pathways of EZH2 activity and underlines the role of PARP1 activity and EZH2 in DNA repair mechanisms. It can thus be postulated that a transition between nuclear soluble and chromatin-bound EZH2 occurs after tissue damage, which indicates a variability of EZH2 expression in time. The aforementioned results may provide an explanation for our findings, as it has been suggested that EZH2 expression depends on the timing and type of DNA-damage and EZH2 also serves a protective function with regard to DNA and tissue regeneration.

According to Chang et al. (19), the silencing of EZH2 correlates with higher expression of E-cadherin and a reduction of N-cadherin and Vimentin. It has been reported that cancer cell invasion correlates with a shift in E-cadherin expression, from membranous to cytoplasmatic (43), and heterotypic E-cadherin/N-cadherin adhesions have been detected in oral SCC and its impairment hinders cancer cell invasion (44). A further potential focus for future studies could therefore be to determinate further correlations of EZH2-expression during cell invasion and even an analysis between primary tumor and LNM. The fraction of cells expressing EZH2 in breast cancer LNM was significantly higher than its primary tumor, while E-cadherin expression in the LNM was lower (45). This could suggest that the comparison of EZH2 expression in primary cSCC and the corresponding LNM may be of interest in future studies.

Upregulation of EZH2 expression has been often detected in injured tissue. Cerulein is used for induction of pancreatitis and therefore impairment of pancreatic tissue. Mallen-St. Clair et al. (46) observed accumulation of EZH2 after pancreatitis induction in mice models, and lack of parenchymal regeneration in mice with EZH2 gene deletion. It has been suggested that lack of EZH2 would lead to failed transcriptional silencing of CDK-Inhibitor 2A (p16) in the PDX1- positive metaplastic lesions, accelerating Kras-driven neoplasia. A similar outcome in terms of acceleration of the Kras-driven lung adenocarcinoma, a shorter lifespan and a higher tumor load has been likewise reported (47). EZH2 suppression enhanced Akt and ERK activation over insulin-like growth factor 1. The authors also described aggravation of inflammatory reaction by macrophage and neutrophil infiltrates and tumor-related IL-6 and TNF-α cytokines. These findings indicate that a cautious approach should be taken with regard to future EZH2-inhibition.

### 4.4 Is the analysis of EZH2-expression useful as a clinical predictive marker in LSCC and ESCC?

One of the most relevant prognostic factors is LNM due to a higher chance of recurrence chances and higher mortality rates. Effective staging to identify LNM would therefore benefit therapy (7, 8, 10, 11). While some authors recommend neck dissection in most cases (48), it is important not to enforce unnecessary overtreatment. The current guidelines for cSCC reflects the need for further safe therapeutical predictors, with no consensus regarding the degree of appropriate lymphadenectomy in the head and neck region, while indicating that the radical neck dissection is not superior to its selective form (7). Elective neck dissection involves risking surgical complications and long-term comorbidities, distresses the patient physically and psychologically, burdens the healthcare system, and does not always carry a benefit (6, 49). Therefore, identifying LNM anticipatively would favor outcomes. Our results show that the analyzed EZH2-score systems are correlated with the degree of spread in locoregional lymph nodes in lip and ear SCC, but cannot predict LN+. Therefore, the incorporation of an EZH2 score purely as an LNM-prediction-model is not apt.

No EZH2-score variant emerges as superior for the analysis of clinical outcome within the matched pairs. Our data showed that a higher EZH2 score was associated with a more undifferentiated tumor grading (score I, II, III), higher AJCC-Stage (score III) and advanced pN-stage (score II, III). These parameters are related to an overall inferior prognosis, and the findings are congruent with the literature on other tumor entities (19, 20, 39). Also, the data of Xie et al. (39) showed the tendency of a greater contrast in EZH2 expression between the G1 and G2 cSCCs, a similar trend as our findings. This could be the result of the affiliation of EZH2 with the cell cycle, cell growth (50), and proliferation (45). The physiological effect of EZH2 on the cell cycle and vice versa is complex and involves epigenetic, biochemical, and clinical parameters (16); future exploration on these effects regarding high-risk SCCs would therefore benefit diagnostics and therapy.

In our study cohort, patients with a lower EZH2 score had a higher risk of local relapse (score I, II, III) and DM (score I, II), being unfavorable for the outcome (**Figure 4**). Our data shows a similar correlation between the EZH2 staining intensity and the occurrence of DM and local relapse (**Figure 9**). A limited number of patients registered a DM, and thus, further research is recommended to elucidate the correlation between low EZH2-score and -intensity and the occurrence of DM with a larger cohort. Additionally, we hypothesize that the degree of staining intensity may impact the ultimate outcomes of the EZH2-score assessments. Therefore, it can be suggested that the occurrence of DM and local relapse is more closely associated with a low staining intensity. These observations could result in closer follow-up care, due to poorer outcomes. Further research, for defining clear cut-off values and analysis of various EZH2-intensity between primary tumor tissue, tumor relapse tissue and distant metastasis would be of clinical interest and could benefit in the future.

Furthermore, no correlation was observed between the extent of the EZH2-staining and clinical outcome, and no difference was identified between the metastasis and non-metastasis groups (**Figures 8**, **9**). Nevertheless, our study revealed that most tumor samples exhibited an EZH2 expression of > 50%. Zhou et al. (25) defined a positive EZH2 expression as > 10% of positive stained cells in tumor tissue. Our data led to the conclusion that the vast majority of cells in high-risk cSCC samples overexpress the enzyme EZH2. A similar matched pairs comparison of the EZH2 expression, while fractioning to distinguish soluble nuclear EZH2 and chromatin-affiliated EZH2, may offer potential benefits for further research. Based on the above-mentioned molecular pathways in section 4.3, we suggest that the predictive value of EZH2 might be improved by simultaneous determination of PARP1-levels.

Taking in consideration the previous stated correlations, these findings would suffice for an individualized prognostic value of EZH2. The analyzed data indicates that EZH2 expression intensity does not increase exponentially in tumor tissue, but is altered during tumor progression, and outcome parameters should be evaluated individually. While high EZH2 scores and its association with poor outcomes may be of therapeutic relevance, the identification of patients with a low EZH2-intensity may also serve as a diagnostic tool in the future. Still, we recommend further analysis for determination a viable cut-off value and further research on the correlations of EZH2 with tumor type, location, progress, and its therapeutic response, before EZH2 diagnostics and therapy makes the transition to routine hospital practice.

### 4.5 Critical handling of EZH2-inhibition

Although many studies have presented EZH2 as an oncogenic factor, a tumor suppressive component has also been acknowledged and studied.

Nienstedt et al. (33) established a prognostic relation between EZH2-overexpression in head and neck cancer and LNM, but did not include cutaneous carcinomas. They also reported a correlation between EZH2-expression and anatomical tumor subsite, but no relevance to differentiation, tumor stage, DM, or patient survival. Banerjee et al. (51) also reported elevated EZH2-levels in HNSCC tissue samples, but did not establish distinctions between EZH2 expression in early and advanced tumor stage. Similar data was reported for laryngeal SCC (28). Some findings have indicated that EZH2 suppresses tumor activity in myeloid malignancies and confirmed the adverse prognostic effects of EZH2-mutations (52, 53). While EZH2-overexpression is associated with prevalence of osteosarcoma, EZH2-knockdown did not prevent osteosarcoma progression (54). Novel studies have reported more aggressive oral SCCs after EZH2-knockdown in a mouse model and discussed the tumor suppressor role of EZH2, concluding that EZH2-expression may differ even in the same cancer entity depending on the stage of disease. It has been suggested that EZH2 has different functions in the timeline of tumor genesis and progression (30).

The data substantiates the findings of Wassef et al. (55), which suggests that overexpression may not be just a causal cancerous factor. It has also been highlighted that EZH2 is rather a result of a high cell proliferation rate and its correlation with poor outcome is mainly linked to the unusually high proliferation. In breast cancer tissue, low EZH2-expression supports cell invasion and tumor growth and is correlated with a poor prognosis. Studies in solid tumor mouse models have indicated that EZH2-expression is not essential for prostate and mammary tumor progression, and they critically question the safety of EZH2 inhibitors. The authors therefore highlight the importance of specific identification of the tumor types which could benefit from targeted therapy (55, 56). Additionally, EZH2 inhibition could also impede physiologic methylation sequences in healthy cells (16). The diversity of our findings suggests that assuming exclusive advantages of EZH2 inhibition is not feasible. Therefore, it is necessary to identify patients who stand to benefit from it.

### 4.6 EZH2 represents a promising potential target for therapeutic applications

EZH2 is an important key in the development and evolution of cancer and therefore a potential therapeutical target. Therefore, supporting chemotherapy or immunotherapy with EZH2-targeting could be effective and beneficial. There is evidence that low EZH2 levels correlate with more favorable chemotherapy results in cisplatin-based therapy in HNSCC (19), and that EZH2 overexpression reduces the cell response to cisplatin therapy in laryngeal SCC (28). A comparable association was observed between EZH2 expression and esophageal SCC chemoradiotherapy response (29). Individual identification of patients who could benefit from improved therapeutic results is of high clinical interest.

Notwithstanding the absence of a correlation between the extent of EZH2 expression and clinical parameters under consideration, it is evident that the majority of the evaluated cSCC tumor tissue samples exhibited EZH2 expression. The data indicated that a higher EZH2-score was linked to a more undifferentiated tumor grading, a higher AJCC-stage, and an advanced pN-stage. These parameters are frequently gathered during the initial diagnosis and tumor staging process. Consequently, if the EZH2-therapy will prove as efficient in cSCC, EZH2 analysis may serve as a viable pre- or perioperative predictor for targeted selection of patients who could benefit from tazemetostat therapy. Our evaluation of EZH2 expression showed a lower score in patients who developed a local relapse and DM. The analysis of EZH2 may also serve as a viable predictor resulting in closer follow-up care for a specific subset of patients. In alignment with previous research, our study provides a foundation for understanding and determining EZH2-expression in high-risk carcinomas. The subsequent phase of the integration of the determination of EZH2-expression in clinical routine would be a randomized controlled trial study. This trial could entail an analysis of the EZH2-score in conjunction with the response rate to tazemetostat and a range of clinical outcome parameters, with the objective of establishing clear cut-off values that can be integrated into clinical practice.

Targeting EZH2 inhibited HNSCC tumor growth and induced cell apoptosis *in vivo* (25). One of the most recent studies on SCC examines EZH2 inhibition with tazemetostat in combination with PD-1 inhibition in patients with recurrent or metastatic HNSCC. Phase 2 of the study is enrolling patients, so the results are yet to be analyzed. Currently, 12 patients have enrolled in Phase 1, and Phase 2 aims to measure the response rate by iRECIST, an objective methodology to evaluate the solid tumors (57). In view of the aforementioned role of PARP1 as regulatory enzyme of EZH2, it would be prudent to consider the potential benefits of additional PARP1-inhibition for future research. A future addition of olaparib, as PARP1 inhibitor to EZH2 inhibition therapy has been also discussed (40) as a promising and clinically compelling approach.

## 5 Conclusion

Current research indicated that EZH2 plays a pivotal role in the progression of cancer. The data available indicate only a low predictive diagnostic benefit in relation to the outcome. Nevertheless, the high expression of EZH2 in the high-risk localization of cSCC suggest that clinical testing of EZH2 inhibition with tazemetostat may be beneficial. The analysis methods presented here thus form the basis for follow-up studies to test EZH2 pre-therapeutically before EZH2 inhibition.

## Data Availability

The raw data supporting the conclusions of this article will be made available by the authors, without undue reservation.
